# Association Between Dietary Pattern Adherence and Blood Pressure Control Among U.S. Adults: A Descriptive Analysis of National Health and Nutrition Examination Survey (NHANES) 2017-2020 Data

**DOI:** 10.7759/cureus.101291

**Published:** 2026-01-11

**Authors:** Akintunde C Akinboboye, Chibuzo C Manafa, Abosede O Odukale-Okuneye, Nonso Ariahu, Ifeoluwa Adesoye

**Affiliations:** 1 Emergency, University of Medical Sciences Teaching Hospital, Ondo, NGA; 2 Family Medicine, Queensland Medical Clinic, Calgary, CAN; 3 Stroke Medicine, Sherwood Forest Hospitals NHS Foundation Trust, Nottinghamshire, GBR; 4 Medicine and Surgery, College of Medicine, University of Lagos, Lagos, NGA; 5 Occupational Health and Safety, Southern Illinois Healthcare (SIH) Harrisburg Medical Center, Harrisburg, USA

**Keywords:** blood pressure, dash diet, dietary patterns, hypertension, nhanes, public health

## Abstract

Background: Hypertension continues to be one of the most pressing health challenges worldwide, contributing significantly to cardiovascular disease and premature death. Dietary approaches aimed at improving blood pressure control have shown promise, yet their effectiveness in real-world settings across diverse populations in the United States is not fully understood.

Objective: To examine, in a cross-sectional analysis, the association between adherence to the Dietary Approaches to Stop Hypertension (DASH) dietary pattern and blood pressure control status among hypertensive U.S. adults using the National Health and Nutrition Examination Survey (NHANES) 2017-2020 data.

Methods: This cross-sectional study analyzed 2,004 unweighted hypertensive adults representing 53,882,767 weighted U.S. adults. DASH adherence was classified into low, moderate, and high categories using the Food Patterns Equivalents Database (FPED). Survey-weighted logistic regression was applied to assess associations between DASH adherence and controlled blood pressure, adjusting for sociodemographic, behavioral, and clinical factors.

Results: No significant association was observed between DASH adherence and blood pressure control (p>0.05). Predicted probabilities of control were 0.45, 0.42, and 0.43 for low, moderate, and high adherence, respectively.

Conclusion: Higher DASH adherence did not significantly improve blood pressure control among U.S. adults with hypertension, highlighting the need for enhanced dietary adherence strategies in real-world settings.

## Introduction

Hypertension is a major health issue affecting the world, being among the most important risk factors of cardiovascular morbidity and mortality in the world [1]. Hypertension is currently estimated to affect up to 47% of adult Americans, and even though effective pharmacologic treatment options exist, blood pressure (BP) management among hypertensive patients is still inadequate, with under half of all hypertensive adults reaching their goal BP [2]. Chronic high BP is one of the major causes of stroke, heart failure, chronic kidney disease, and early cardiac death [3,4]. Lifestyle and dietary change is, therefore, one of the pillars of hypertension prevention and treatment, which can be used along with pharmacologic interventions [5]. Among other dietary measures, a nutritional intervention based on compliance with the Dietary Approaches to Stop Hypertension (DASH) pattern has become one of the most effective measures in BP management [6,7].

The DASH dietary pattern was first created in the 1990s and includes high fruits, vegetables, whole grains, low-fat dairy, nuts, and lean proteins, and has a small quantity of sodium, saturated fats, and added sugars [8]. Clinical and population-based investigations have continuously indicated that the DASH diet results in meaningful decreases in systolic and diastolic BP in hypertensive and normotensive persons [9,10]. In addition, compliance with DASH has been linked to reduced cases of cardiovascular diseases, metabolic syndrome, and all-cause mortality [11]. Although the evidence provided by controlled trials is strong, it is a difficult task to translate the findings into actual adherence, especially when it comes to diverse populations within the U.S., where socioeconomic, cultural, and behavioral influences can affect dietary habits and access to healthy foods [12,13].

Although the DASH diet is shown to be effective in controlled conditions, the evidence on its effectiveness at the population level regarding the control of actual BP in hypertensive adults in recent years is rather scarce [14]. Notably, with alterations in dietary practices, food environments, and revised guidelines on hypertension, this association should be reconsidered using recent national data [15,16].

The study will utilize National Health and Nutrition Examination Survey (NHANES) 2017-2020 data in exploring the association between dietary pattern adherence and BP control in U.S. adults. NHANES offers rich information on nutritional habits, lifestyle habits, and clinical outcomes, enabling investigators to determine the extent to which the diets of individuals follow evidence-based dietary plans such as DASH [17]. This void implies that although there is solid clinical evidence that DASH is a non-pharmacologic intervention with substantial success in the treatment of hypertension, its practical application is scarce [18,19]. The knowledge of the relationship between DASH compliance and BP in modern American populations is thus of vital concern to the planning and development of policies in the field of health matters [20].

The objective of the study is to assess the association between adherence to the DASH pattern and BP control status among adults with hypertension. This study will advance the knowledge about the effects of dietary patterns in hypertension management in real-life conditions and inform further dietary recommendations and interventions that promote cardiovascular health among people by quantifying this relationship in a nationally representative sample.

## Materials and methods

Study design and data source

This cross-sectional study used publicly available data from the NHANES 2017-March 2020 (pre-pandemic) cycles [21]. NHANES is a continuous, nationally representative survey of the civilian noninstitutionalized U.S. population that combines household interviews with standardized mobile examination center (MEC) assessments. For dietary pattern measurement, we used the Food Patterns Equivalents Database (FPED) release prepared for the What We Eat in America (WWEIA)/NHANES 2017-March 2020 to translate 24-hour recall data into food-group equivalents; these FPED food-group variables were linked to NHANES participant records and averaged across available day-1 and day-2 recalls to better represent usual intake [22]. The analytic dataset created for this study merged the FPED mean intake file with NHANES demographic, BP examination (oscillometric readings), hypertension, body measures, alcohol, smoking, physical activity, and diabetes using the sequence number (SEQN) variable that represents respondents in the survey.

Study population

The target population comprised U.S. adults(≥ 20 years) with hypertension identified within the study period, 2017-March 2020. Hypertension status for inclusion was defined as self-report of a physician diagnosis of high BP, current antihypertensive medication use, or measured mean systolic BP ≥130 mmHg or mean diastolic BP ≥80 mmHg based on up to three oscillometric readings taken in the MEC. Participants were restricted to adults who met the hypertension definition and for whom complete data were available on the exposure, outcome, and covariates used in the main models; after applying complete-case criteria and the hypertension restriction, the analytic sample consisted of 2,004 unweighted participants representing 53,882,767 weighted U.S. adults.

Variables/measures

The primary exposure of interest was adherence to the DASH dietary pattern, operationalized as a seven-component food-group score derived from FPED food-group equivalents. Sodium intake was not included because the FPED-based scoring captures food-group intake rather than nutrient-level intake, aligning with prior NHANES-based DASH analyses. Components included fruits, vegetables, whole grains, low-fat dairy, nuts/legumes, red/processed meats (reverse-scored), and added sugars (reverse-scored). Component intakes were averaged across day-1 and day-2 dietary recalls and expressed in FPED equivalents. Quintiles were computed for each component using Stata’s xtile command (StataCorp LLC, College Station, Texas, USA), with reverse scoring applied for meats and added sugars. Total DASH scores (range 7-35) were calculated by summing component scores and categorized as low, moderate, or high adherence (≤17=low, 18-24=moderate, ≥25=high).

The primary outcome was BP control among hypertensive adults, defined as mean systolic pressure <130 mmHg and mean diastolic pressure <80 mmHg, calculated from up to three oscillometric readings taken in the MEC. Key covariates included age, gender, race/ethnicity, poverty-income ratio, education, lifestyle behaviors (smoking, alcohol use, and moderate-to-vigorous physical activity), and diabetes status.

All NHANES files were merged using the respondent identifier (SEQN), and survey weights (WTMECPRP), strata (SDMVSTRA), and primary sampling units (PSU) (SDMVPSU) were applied to account for the complex, multistage sampling design. This approach allows nationally representative estimates of DASH adherence and BP control.

Statistical analysis

All analyses accounted for the complex, multistage probability sampling design of NHANES by using survey procedures provided by NHANES. Descriptive statistics were conducted as survey-weighted means and proportions for the analytic sample restricted to adults with hypertension. Because this analysis uses complex survey design data to produce nationally representative estimates, we employed survey-design-based F-tests (Rao-Scott adjusted F-tests) to compare groups for categorical variables, and survey-adjusted t-tests for continuous variables. The primary multivariable analysis modeled the association between DASH adherence as a categorical variable and the binary outcome of BP control using survey-weighted logistic regression adjusted for sociodemographic, behavioral, and clinical covariates. Multicollinearity was assessed using the variance inflation factor (VIF), which ranged between 1.06 and 4.40, with a mean VIF of 1.83. All statistical testing used a two-sided alpha of 0.05, and analyses were performed using Stata version 18 (StataCorp LLC, College Station, TX, USA) [23].

Missing data

Missingness was evaluated for all variables used in the analysis. Because the data derive from a complex, multistage survey with design-based weights, multiple imputation procedures that do not appropriately account for survey design features may introduce bias; therefore, imputation methods were not applied for the main analysis. Instead, a complete-case approach was used, retaining participants with non-missing values on the exposure (DASH score), outcome (BP control), and covariates. Overall, missingness was 13.22% for BP control and 18.49% for the DASH adherence score.

Ethical considerations

This study used de-identified, publicly available data from NHANES and did not require additional institutional review board approval. The original NHANES protocol was approved by the National Center for Health Statistics Research Ethics Review Board, and all participants provided informed consent at the time of data collection.

## Results

Table 1 presents the weighted demographic, behavioral, and clinical characteristics of U.S. adults with hypertension, stratified by BP control status using data from NHANES 2017-2020. The analysis reflects an estimated 53,882,767 weighted adults and incorporates the complex multistage survey design to ensure national representativeness.

**Table 1:** Weighted characteristics of U.S. adults with hypertension by BP control status, NHANES 2017-2020. The category “≥5 days of moderate-to-vigorous physical activity” contained no observations following complete case analysis and was therefore excluded (recorded as NA). Values are weighted means ± standard deviations (SDs) for continuous variables and weighted counts (n) with percentages (%) for categorical variables. All estimates are survey-weighted to represent the civilian, noninstitutionalized U.S. population. Differences between groups were evaluated using survey-adjusted t-tests and Rao-Scott F-tests. Analyses were conducted using Stata version 18 [23]. GED: general education development; PA: physical activity; DASH: Dietary Approaches to Stop Hypertension; BP: blood pressure; NHANES: National Health and Nutrition Examination Survey

Characteristic	Controlled BP (N=23,101,899)	Uncontrolled BP (N=30,780,868)	F-test/T-test	P-value
Age in years (mean±SD)	58.06±13.82	58.45±15.57	t=0.49	0.630
Income-to-poverty ratio (mean±SD)	3.32±1.49	3.00±1.66	t=-2.18	0.039
Mean systolic BP in mmHg (mean±SD)	115.29±9.09	141.76±16.40	t=28.76	<0.001
Mean diastolic BP in mmHg (mean±SD)	68.61±7.37	83.30±11.89	t=22.19	<0.001
DASH score (mean±SD)	20.87±3.99	20.70±4.46	t=-0.67	0.510
DASH score (n, %)			F=0.159	0.848
Low adherence	5,279,986 (43)	7,118,168 (57)		
Moderate adherence	13,102,184 (44)	17,777,896 (58)		
High adherence	4,719,729 (45)	5,884,804 (55)		
Gender (n, %)			F=0.04	0.850
Male	11,750,950 (43)	15,478,248 (57)		
Female	11,350,949 (43)	15,302,620 (57)		
Education level (n, %)			F=7.92	0.003
Less than high school	1,580,839 (28)	4,053,454 (72)		
High school graduate/GED	7,172,484 (43)	9,590,075 (57)		
College or higher	14,348,575 (46)	17,137,339 (54)		
Diabetes (n, %)			F=0.001	0.940
Diabetes	5,865,486 (43)	7,755,920 (57)		
No diabetes	17,236,412 (43)	23,024,948 (57)		
Smoking (n, %)			F=1.07	0.345
Never	11,506,215 (43)	15,194,211 (57)		
Former	8,333,463 (45)	10,131,556 (55)		
Current	3,262,220 (37)	5,455,101 (63)		
Moderate to vigorous PA (n, %)			F=0.004	0.950
1-2 days	4,856,688 (43)	6,557,386 (57)		
3-4 days	18,245,211 (43)	24,223,482 (57)		
≥5 days	NA	NA	NA	NA
Alcohol use (n, %)			F=3.31	0.081
Yes	22,243,827 (44)	28,739,486 (56)		
No	858,072 (30)	2,041,382 (70)		
Race/ethnicity (%)			F=7.71	<0.001
Mexican American individuals	865,947 (36)	1,539,718 (64)		
Other Hispanic individuals	1,467,167 (42)	2,016,518 (58)		
Non-Hispanic White individuals	18,469,254 (46)	22,084,714 (54)		
Non-Hispanic Black individuals	2,299,531 (31)	5,139,918 (69)		

Among 2,004 hypertensive adults, representing 53,882,767 individuals nationwide, approximately 23,101,899 (43%) had controlled BP, while 30,780,868 (57%) had uncontrolled BP. The mean age was comparable between groups (58.1±13.8 years vs. 58.5±15.6 years, p=0.63). Participants with controlled BP had a slightly higher mean income-to-poverty ratio (3.32±1.49) than those with uncontrolled BP (3.00±1.66, p=0.039). As expected, mean systolic and diastolic BP values were significantly lower among individuals with controlled hypertension (115.3±9.1 mmHg and 68.6±7.4 mmHg) compared with their uncontrolled counterparts (141.8±16.4 mmHg and 83.3±11.9 mmHg; both p<0.001). Average DASH adherence scores did not differ significantly between groups (p=0.51).

Further, sociodemographic patterns indicated higher BP control among participants with higher education: 14,348,575 (46%) of those with a college education had controlled BP versus 1,580,839 (28%) among those with less than a high-school education (F=7.92, p=0.003). Race/ethnicity differences were also evident (F=7.71, p<0.001), with non-Hispanic White adults showing the highest prevalence of BP control (18,469,254, 46%) and non-Hispanic Black adults the lowest (2,299,531, 31%). No significant associations were observed between BP control and diabetes status (p=0.94), smoking (p=0.35), physical activity (p=0.95), or alcohol use (p=0.08).

Table 2 presents the unadjusted association between DASH diet adherence and BP control among hypertensive U.S. adults.

**Table 2:** Crude association between DASH adherence and blood pressure control among U.S adults with hypertension, NHANES 2017-2020. Odds ratios (ORs) and 95% confidence intervals (CIs) were estimated using survey-weighted logistic regression to account for NHANES’ complex sampling design. The reference group for DASH adherence was “Low.” Estimates are weighted to represent 53,882,767 U.S. adults with hypertension during 2017-2020. Analyses were conducted using Stata version 18 [23]. NHANES: National Health and Nutrition Examination Survey; DASH: Dietary Approaches to Stop Hypertension

Predictor	OR (95% CI)	P-value
DASH adherence category		
Moderate vs. low	0.99 (0.73-1.35)	0.966
High vs. low	1.08 (0.73-1.60)	0.683

From the crude analyses above, it’s evident that neither moderate nor high DASH adherence was significantly associated with improved BP control compared to low adherence (OR=0.99, 95% CI: 0.73-1.35, p=0.97; and OR=1.08, 95% CI: 0.73-1.60, p=0.68, respectively). These findings suggest that without adjustment for demographic and behavioral factors, DASH adherence alone was not significantly linked to BP control in this population.

Table 3 presents an adjusted model for demographic, behavioral, and clinical characteristics.

**Table 3:** Adjusted association between DASH adherence and blood pressure control among U.S. adults with hypertension, NHANES 2017-2020. Survey-weighted multivariable logistic regression was used to examine the adjusted association between DASH adherence and blood pressure control among U.S. adults with hypertension (n=2,004; weighted N=53,882,767). The model controlled for sociodemographic (age, gender, race/ethnicity, income-to-poverty ratio, education), behavioral (physical activity, smoking, alcohol use), and clinical (diabetes) factors. Analyses were conducted using Stata version 18 [23]. NHANES: National Health and Nutrition Examination Survey; DASH: Dietary Approaches to Stop Hypertension; GED: general education development; MVPA: moderate-to-vigorous physical activity

Predictor	Odds Ratio (95% CI)	P-value
DASH adherence category		
Moderate vs. low	0.88 (0.64-1.22)	0.436
High vs. low	0.92 (0.62-1.37)	0.669
Age (years)	1.00 (0.99-1.00)	0.253
Gender (female vs. male)	1.09 (0.82-1.46)	0.541
Race/ethnicity		
Other Hispanic vs. Mexican American individuals	1.28 (0.75-2.19)	0.357
Non-Hispanic White vs. Mexican American individuals	1.27 (0.83-1.94)	0.261
Non-Hispanic Black vs. Mexican American individuals	0.73 (0.43-1.25)	0.245
Income-to-poverty ratio (PIR)	1.07 (0.94-1.23)	0.295
Education		
High school graduate/GED	1.74 (1.25-2.44)	0.002
College or higher	1.77 (1.25-2.50)	0.002
Physical activity (MVPA)		
3-4 days/week vs. <3 days	1.03 (0.59-1.78)	0.924
Smoking status		
Former vs. never	1.05 (0.74-1.51)	0.764
Current vs. never	0.84 (0.55-1.29)	0.413
Alcohol use (yes vs. no)	1.67 (0.80-3.50)	0.164
Diabetes (yes vs. no)	1.10 (0.84-1.43)	0.481

After adjusting for demographic, behavioral, and clinical characteristics, neither moderate nor high adherence to the DASH diet was significantly associated with higher odds of BP control compared to low adherence (OR=0.88, 95% CI: 0.64-1.22; OR=0.92, 95% CI: 0.62-1.37, respectively). Age, gender, race/ethnicity, and lifestyle factors were not significant predictors. However, participants with higher educational attainment showed significantly greater odds of BP control; those with a high school or college education had roughly 1.7 times higher odds compared to adults with less than a high school education (p=0.002). These findings suggest that while DASH adherence alone was not independently linked to BP control, education remained an important determinant in this population.

**Figure 1:**
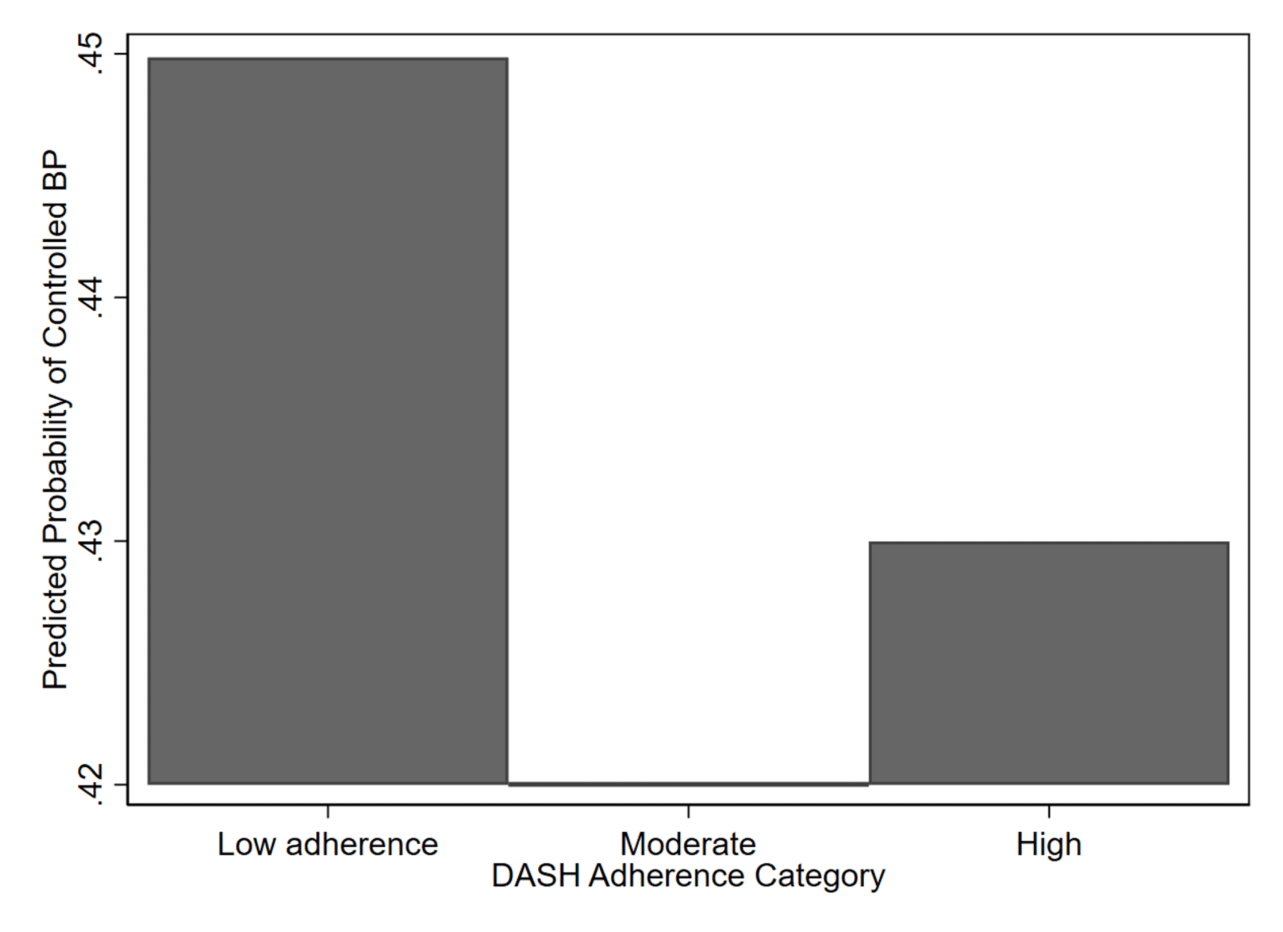
Predicted probability of controlled blood pressure according to DASH adherence category among U.S. adults with hypertension, NHANES 2017-2020. Estimates were derived from survey-weighted logistic regression models adjusted for sociodemographic, behavioral, and clinical factors (age, sex, race/ethnicity, income-to-poverty ratio, education, physical activity, smoking, alcohol use, and diabetes). Error bars are not displayed because the predicted probability differences were statistically nonsignificant (p > 0.05). Generated using Stata version 18 [23]. DASH: Dietary Approaches to Stop Hypertension; NHANES: National Health and Nutrition Examination Survey

Figure 1 presents the adjusted predicted probabilities of achieving BP control across categories of DASH diet adherence among hypertensive U.S. adults.

As shown in Figure 1, the predicted probability of achieving BP control was similar across DASH adherence levels, with participants in the low adherence group exhibiting the highest predicted probability, approximately 0.45, compared to moderate and high adherence groups (between 0.42 and 0.43). Despite the expected inverse trend, these differences were not statistically significant after adjustment for potential confounders. This finding suggests that in the 2017-2020 U.S. hypertensive population, adherence to the DASH dietary pattern was not independently associated with BP control when accounting for lifestyle and clinical factors.

## Discussion

In this nationally representative sample of hypertensive U.S. adults from NHANES 2017-2020, there was no significant association between adherence to the DASH dietary pattern and BP control after accounting for sociodemographic, behavioral, and clinical characteristics. The strong independent association observed between education and BP control suggests that social context may play an important role in shaping the effectiveness of dietary patterns such as DASH. Higher educational attainment may facilitate better nutrition knowledge, food access, and sustained adherence, whereas structural and socioeconomic constraints may limit the translation of dietary recommendations into effective BP control among lower-education groups. Although individuals with higher DASH adherence demonstrated slightly lower predicted probabilities of achieving BP control, these differences were small and not statistically meaningful. These findings contrast with the substantial BP-lowering effects reported in controlled clinical trials, where the DASH diet consistently reduced systolic and diastolic pressures across diverse populations [6-9].

A key explanation for this discrepancy is the challenge of translating structured dietary interventions into real-world behavior. Clinical trials typically provide intensive counseling, monitored food intake, and controlled sodium levels, conditions that facilitate adherence and amplify physiological benefits. In contrast, adherence in community settings is influenced by socioeconomic and environmental constraints, including variable access to fresh produce, food cost, neighborhood food environments, and cultural food preferences [12,13,18]. These constraints may diminish the likelihood that adults who appear to follow a DASH-consistent pattern in NHANES actually achieve the dietary quality, sodium restriction, and food balance necessary to replicate trial-level effects.

Moreover, the biological mechanisms through which diet influences arterial pressure may not be fully engaged if individuals simultaneously experience competing lifestyle factors. Sodium and potassium intake exert well-established effects on intravascular volume, vascular tone, neurohormonal activation, and endothelial function. Excess sodium promotes sympathetic activation, renin-angiotensin-aldosterone system (RAAS) stimulation, and arterial stiffness, while potassium-rich diets, central to both DASH and Mediterranean patterns, facilitate natriuresis and vascular relaxation by increasing nitric oxide synthesis [9,23]. Although DASH adherence scores capture major food-group intakes, they do not directly quantify sodium consumption or account for behaviors that counteract dietary benefits, such as physical inactivity, weight gain, and inconsistent medication use [14-16]. Thus, even individuals categorized as high DASH adherers may not reach the nutrient thresholds needed to influence arterial physiology meaningfully.

Prior cross-sectional studies using earlier NHANES cycles have also yielded heterogeneous findings, with some reporting modest associations only in specific subgroups [14,15]. These study results are consistent with this variability and reinforce the interpretation that dietary quality alone is unlikely to be a sufficient driver of BP control at the population level. This is supported by contemporary American Heart Association/American College of Cardiology (AHA/ACC) guidelines, which emphasize that dietary interventions such as DASH are most effective when embedded within a broader, holistic lifestyle approach that includes physical activity, weight management, sodium restriction, and pharmacologic therapy when indicated [5]. Evidence increasingly demonstrates that comprehensive lifestyle patterns, not diet in isolation, produce the most meaningful improvements in BP and cardiovascular risk reduction.

The challenges surrounding dietary adherence also reflect broader structural and socioeconomic determinants of health. Real-world barriers, including the high cost of nutrient-dense foods, limited availability of quality DASH-aligned food items in low-resource communities, cultural dietary preferences, and household food dynamics, may limit sustained adherence, even among motivated individuals [12,13,18]. These barriers may help explain why the biological effectiveness of DASH, well-documented in controlled settings, has not fully translated to population-level BP control.

Despite the lack of significant association in this analysis, the broader relevance of DASH should not be minimized. The dietary pattern remains strongly linked to reductions in cardiovascular disease, metabolic risk, and all-cause mortality [11,17,20]. Therefore, the public health priority may not be to reconsider DASH as a therapeutic strategy, but rather to strengthen the social and environmental supports necessary for individuals to adopt and sustain it. Community-based interventions, food-cost subsidies, culturally tailored nutrition programs, and policies that improve the affordability and accessibility of nutrient-dense foods may enhance the real-world impact of DASH and similar dietary patterns.

Strengths and limitations

The study’s strengths include the use of recent nationally representative NHANES data, standardized BP measurements, and adjustment for multiple confounders. An important limitation of this cross-sectional analysis is the inability to establish temporality between dietary adherence and BP control. Reverse causation is plausible, as individuals with uncontrolled hypertension or a recent diagnosis may adopt healthier dietary patterns, including DASH-aligned behaviors, in response to medical advice. Such post-diagnosis dietary modification could bias associations toward the null and partially explain the lack of observed benefit of higher DASH adherence in this population-based analysis. However, the pre-COVID-2017-2020 sample limited the inclusion of more recent trends in dietary behavior and healthcare access. Additionally, dietary intake was self-reported, which may introduce recall bias. Future research should examine post-pandemic data to assess evolving dietary patterns and explore longitudinal designs that better capture sustained DASH adherence and its impact on hypertension management across diverse U.S. populations.

## Conclusions

This study found no significant association between adherence to the DASH dietary pattern and BP control among U.S. adults with hypertension in NHANES 2017-2020. These findings suggest that, in real-world settings, factors such as variable adherence, broader lifestyle behaviors, and medication use may influence observed outcomes. The results highlight the gap between dietary recommendations and actual practice and underscore the importance of comprehensive hypertension management strategies that integrate sustainable dietary changes with medical and behavioral interventions.
